# RIPK3-Dependent Necroptosis Limits PRV Replication in PK-15 Cells

**DOI:** 10.3389/fmicb.2021.664353

**Published:** 2021-06-04

**Authors:** Hongchao Gou, Zhibiao Bian, Rujian Cai, Pinpin Chu, Shuai Song, Yan Li, Zhiyong Jiang, Kunli Zhang, Dongxia Yang, Chunling Li

**Affiliations:** ^1^Institute of Animal Health, Guangdong Academy of Agricultural Sciences, Guangzhou, China; ^2^Guangdong Provincial Key Laboratory of Livestock Disease Prevention, Guangzhou, China; ^3^Maoming Branch, Guangdong Laboratory for Lingnan Modern Agriculture, Maoming, China; ^4^Scientific Observation and Experiment Station of Veterinary Drugs and Diagnostic Techniques of Guangdong Province, Guangzhou, China

**Keywords:** pseudorabies virus (PRV), necroptosis, receptor interacting protein kinase 3 (RIPK3), mixed lineage kinase-like protein (MLKL), PK-15 cell

## Abstract

Pigs infected by pseudorabies virus (PRV) display necrotic pathology in multiple organs. The mechanism by which PRV induces cell death is still unclear. Recently, necroptosis was identified as a programmed process dependent on the receptor interacting protein kinase 3 (RIPK3) and mixed lineage kinase-like protein (MLKL). In this study, we demonstrated that PRV induced RIPK3-dependent necroptosis in PK-15 cells. The data showed that PRV infection caused cell death with Propidium Iodide (PI)-positive staining. Transmission electron microscopy analysis indicated plasma membrane disruption in PRV-infected cells. A pan-caspase inhibitor did not prevent PRV-induced necrotic cell death. Western blot analysis indicated that caspase-3 and caspase-8 were not cleaved during PRV infection. Although the transcription of tumor necrosis factor-alpha (TNF-α) was increased by PRV infection, RIPK1 was shown to be not involved in PRV-induced necrotic cell death by use of its specific inhibitor. Further experiments indicated that the phosphorylation of RIPK3 and MLKL was upregulated in PRV-infected cells. Stable shRNA knockdown of RIPK3 or MLKL had a recovery effect on PRV-induced necrotic cell death. Meanwhile, viral titers were enhanced in RIPK3 and MLKL knockdown cells. Hence, we concluded that initiation of necroptosis in host cells plays a limiting role in PRV infection. Considering that necroptosis is an inflammatory form of programmed cell death, our data may be beneficial for understanding the necrotic pathology of pigs infected by PRV.

## Introduction

Pseudorabies virus (PRV), also known as Aujeszky’s disease virus or Suid herpesvirus type 1 (SuHV-1), is an enveloped virus with a 143 kb double-stranded linear DNA encoding more than 70 proteins (Klupp et al., 2004). The virus belongs to the family Herpesviride that includes herpes simplex virus 1 (HSV-1) and human cytomegalovirus (HCMV) (Davison, 2010). PRV is an infectious agent of disease in multiple mammals, including pigs, ruminants, carnivores, rodents, and so on (Sun et al., 2016). Humans are also reported to be infected by PRV (Li et al., 2020; Liu et al., 2020). Depending on the ability of latent infection and reactivation in trigeminal nerves in pigs, PRV mainly circulates in porcine herds and induces severe economic losses (Rziha et al., 1986). Pathological features in pigs infected by PRV include necrosis in multiple organs, including tonsil, lung, cerebellum, lymph nodes, kidney, and liver (Verpoest et al., 2016; Yang et al., 2016). During the infection in pigs, PRV primarily infects the epithelial cells and crosses the basement membrane in order to infect all cell types in underlying tissues in a necrotic fashion (Nauwynck et al., 2007). *In vitro*, PRV caused cell death of primary porcine epithelial kidney, superior cervical ganglion, and testicle cells (Geenen et al., 2005, 2007). However, the mechanism by which PRV induces cell death is still unclear.

Traditionally, necrosis has been considered as a form of accidental cell death. Recently, necroptosis was found to be a kind of programmed process dependent on the receptor interacting protein kinase 3 (RIPK3) and mixed lineage kinase-like protein (MLKL) (Sun et al., 2012). MLKL targets the cell membrane and destroys its integrity, leading to the release of cellular contents and subsequent inflammatory reactions (Wang H et al., 2014). Tumor necrosis factor-alpha (TNF-α) is a classical inducer of necroptosis *via* death receptors and the cascade reaction of the receptor interacting protein kinase 1 (RIPK1), RIPK3, and MLKL (Holler et al., 2000; Wu et al., 2014). Interestingly, necroptosis can also be activated through non-classical pathways independent of RIPK1. It has been demonstrated that RIPK3 can be initiated in an RIPK homotypic interaction motif (RHIM)-dependent manner. For example, some pattern recognition receptors (PRRs), including DNA-induced activator of interferon (DAI, also known as DLM and ZBP1), Toll-like receptors (TLRs), and retinoic acid-inducible gene 1 (RIG-I) like receptors, directly bind with the RHIM of RIPK3 and initiate the necroptosis pathway (Kaiser et al., 2013; Thapa et al., 2016; Schock et al., 2017).

As an inflammatory form of programmed cell death, necroptosis plays an important role in fighting against viral infection. *Vaccinia* virus-induced tissue necrosis and inflammation is connected with RIPK1-RIPK3 necrosomes. Strikingly, the replication and mortality from *Vaccinia* virus are increased in mice with RIPK3 deletion (Cho et al., 2009). In order to sustain replication in host cells, murine cytomegalovirus (MCMV) infection prevents RIPK3 activity by encoding the RHIM-containing protein M45/vIRA (Upton et al., 2012). Similar to MCMV, HSV-1 has the ability to inhibit necroptosis *via* the interaction of ribonucleotide reductase large subunit (ICP6) with RIPK3 in human cells (Guo et al., 2015b). However, it has been reported that ICP6 is a direct activator of RIPK3 in mouse cells, underlining the species differences in necroptosis pathways (Wang X et al., 2014). Although PRV is closely related to HSV-1, the role of necroptosis in cell death induced by PRV infection is still unclear.

Herein, we explored PRV-induced RIPK3-dependent necroptosis in PK-15 cells. Our data showed that PRV infection caused cell death with necrotic characteristics. Activities of caspase proteins were not involved in PRV-induced necrotic cell death. In addition, classical RIPK1-dependent necroptosis was not related to PRV infection, although the expression of TNF-α was increased. Furthermore, we found that the phosphorylation of RIPK3 and MLKL was upregulated in PRV-infected cells. Stable shRNA knockdown of RIPK3 or MLKL could promote cell viability and enhance viral titers during PRV infection, indicating the limiting role of necroptosis in PRV infection.

## Materials and Methods

### Cells and Viruses

The swine kidney cell line PK-15 (ATCC, CCL-33) was cultured in Dulbecco’s modified Eagle medium (DMEM) (11965; Gibco) supplemented with 10% fetal bovine serum (FBS) (Biological Industries, United States) at 37°C with 5% CO_2_. The classical PRV Rong A (RA) strain was purchased from the China Veterinary Culture Collection Center (CVCC Number: AV25). The currently circulating variant of virulent PRV GD-WH strain (GenBank No. KT948051) was isolated from the brain of a pig suspected to be infected with PRV in 2015. Pure virus was obtained *via* several rounds of plaque purification (Gou et al., 2020).

### Biochemical Reagents and Antibodies

The pan-caspase inhibitor Z-VAD-FMK (S7023) and RIPK1 inhibitor necrostatin-1 (S8037) were purchased from Selleck Chemicals. TNF-α (RP0080S) was the product of Kingfisher Biotech. Staurosporine (S1882) and SM-164 (SC0114) were obtained from Beyotime. The primary antibodies used in this study were as follows: rabbit polyclonal Caspase-3 (AC030; Beyotime), rabbit polyclonal Caspase-8 (AC056; Beyotime), rabbit polyclonal RIPK3 (AF7893; Beyotime), rabbit polyclonal MLKL (A5579; ABclonal), rabbit polyclonal TNF-α (A0277; ABclonal), rabbit polyclonal FasL (A0234; ABclonal), rabbit polyclonal TraiL (A12312; ABclonal), mouse monoclonal GAPDH (AG019; Beyotime), rabbit monoclonal (EPR9627) RIP3 (phospho S227) (ab209384; Abcam), rabbit monoclonal (EPR9514) to MLKL (phospho S358) (ab187091; Abcam), and mouse monoclonal anti-PRV gE (kindly provided by Dr. Gaiping Zhang, College of Veterinary Medicine, Henan Agricultural University, China). The secondary antibodies used for Western blot were HRP-conjugated goat anti-mouse IgG (BS12478; Bioworld Technology) and HRP-conjugated goat anti-rabbit IgG (BS13278; Bioworld Technology).

### Virus Titers Assay

Virus titers were determined on PK-15 cells cultivated in 96-well plates. Virus supernatant was 10-fold serial diluted with DMEM containing 2% FBS. After being inoculated on a cell monolayer and cultured at 37°C for 3 days, cytopathic effects were observed and calculated as the 50% tissue culture infectious dose (TCID_50_) per milliliter according to the Spearman–Karber method (Spearman, 1908; Kärber, 1931).

### Biochemical Intervention

To stimulate apoptosis, PK-15 cells at 80% confluence were treated with 50 nM staurosporine, a protein kinase inhibitor. To prevent apoptosis, 10 μM Z-VAD-FMK was added to the cell medium. To activate TNF-α-induced necroptosis, PK-15 cells were treated with 10 ng/ml TNF-α combined with 2 μM SM-164 and 10 μM Z-VAD-FMK. For inhibiting RIPK1 activities, PK-15 cells were treated with 100 μM necrostatin-1.

### Cell Viability Assay

Cell viability was analyzed by using a Cell Counting Kit-8 (CCK8) assay kit (C0037; Beyotime) according to the manufacturer’s instructions. A seeding density of 1 × 10^4^ cells was cultured in 96-well culture plates for 24 h. Then, the cell monolayer was infected with PRV or stimulated with biochemical reagents. After 24 h of cultivation, cells were washed twice with DMEM and cultured in 100 μl of DMEM containing 10 μl of CCK8 for 1 h. The optical density was measured at 570 nm by using a model 680 microplate reader (Bio-Tek).

### Fluorescent Dye Staining of Apoptotic and Necrotic Cells

PK-15 cells in 96-well culture plates were treated with PRV or biochemical reagents. The cell medium was discarded after 12 or 24 h. The cell monolayer was stained with Hoechst-33342 or Propidium Iodide (PI) solutions (C1056-3; Beyotime) for 10 min at 4°C. According to the manufacturer’s instructions, apoptotic cells stained with Hoechst-33342 will show a bright blue color. Meanwhile, necrotic cells stained with both Hoechst-33342 and PI will show bright blue and red colors. Live images were recorded by using EVOS FL Auto (Life Technologies).

### Flow Cytometry Analysis of Apoptotic Cells

Apoptosis of the cells was analyzed by using the Alexa Fluor^TM^ 488 Annexin V/Dead Cell Apoptosis Detection Kit I (2208491; Invitrogen) according to the manufacturer’s instructions. Briefly, PK-15 cells in 6-well culture plates were dispersed by trypsin. Then, cells were collected by centrifugation at 800*g* for 2 min. After washing twice with PBS, cells were stained with Alexa Fluor 488^TM^ Annexin V and PI solutions for 15 min at room temperature. Flow cytometry analysis was performed on a CytoFLEX (BECKMAN COULTER).

### Transmission Electron Microscopy

The cell monolayers in 10-cm dishes were washed twice with PBS and fixed with 2.5% glutaraldehyde diluted in PBS at 4°C for 30 min. PK-15 cells were scraped and placed into 1.5-ml Eppendorf tubes. Cell pellets were dehydrated with an acetone series and embedded in epoxy resin. Then, ultrathin sections were prepared and observed by using a JEM-2010HR TEM (JEOL).

### Western Blot

Cells were washed with ice-cold PBS and dissolved in radioimmunoprecipitation assay (RIPA) lysis buffer (P0013B; Beyotime) containing 1 mM phenylmethanesulfonyl fluoride (PMSF, ST506; Beyotime) for 10 min. After centrifugation at 14,000*g* at 4°C for 10 min, the protein supernatant was obtained, and its concentration was quantified by using a bicinchoninic acid (BCA) protein assay kit (23227; Thermo Fisher). Twenty micrograms of protein was separated in 12% SDS-PAGE gels and then electro-transferred to polyvinylidene fluoride (PVDF) membranes (IPVH00010; Millipore). PVDF membranes were blocked in PBS containing 5% nonfat milk and 0.1% Tween 20 for 1 h at 25°C. The corresponding primary antibodies were incubated at 4°C for 12 h, and the secondary antibodies conjugated to HRP were incubated at 37°C for 1 h. Then, protein blot signals were amplified by using an ECL Plus kit (P0018FS; Beyotime) and imaged by a chemiluminescence imaging system (Fine-do X6; Tanon). The intensity of protein blots was measured with ImageJ software (Gassmann et al., 2009).

### Construction of PK-15 Cells Stably Expressing shRNA

The shRNAs, respectively, targeting RIPK3 (GenBank No. MG543992.1) and MLKL (GenBank No. MG543991.1) were designed by Cyagen. The shRNA sequences used in this study were as follows. shRIPK3 sequence: ACAGCAACTACATGGTTATAA and shMLKL sequence: ACGAGCTTCCTGGTCACTAAA. To generate stable gene knockdown in PK-15 cells, shRNAs targeted to RIPK3, MLKL, and Scramble shRNAs were transfected into cells by using Lipofectamine 3000 (L3000001; Thermo Fisher). The medium was changed to fresh complete medium containing 1 μg/ml puromycin (ST551; Beyotime) after 24 h post-transfection. After 7 days, live cells were dispersed and diluted to single cells in 96-well culture plates. Then, single cell clones were harvested, and mRNA of each clone was evaluated by reverse transcription-quantitative polymerase chain reaction (RT-qPCR). Finally, the single clone with the lowest mRNA level was used as a gene knockdown cell line.

### RT-qPCR

Total RNA was purified by using a Total RNA Kit I (R6834-01; Omega) according to the manufacturer’s instructions. Complementary DNAs (cDNAs) were synthesized by using PrimeScript^TM^ RT Master Mix (RR036A; TAKARA). Primers of RT-qPCR are listed in Table 1. Using SYBR Premix (RR820A; TAKARA), RT-qPCR was performed on a LightCycler 480 detection system (Roche).

**TABLE 1:** Primer sequences used for RT-qPCR assay.

Gene name	Forward primer (5′-3′)	Reverse primer (5′-3′)
GAPDH	TGGAGTCCACTGGTGTCTTCAC	TTCACGCCCATCACAAACA
TNF-a	TGGCCCAAGGACTCAGATCAT	TCGGCTTTGACATTGGCTACA
FasL	AAGAAGAAGAGGGACCACAATG	CTTTGGCTGGCAGACTCTCT
TraiL	GGAACGGTTTCTACAGAAGGG AAC	TCAGCAGTATAGGGTCAGGA TAGC
RIPK3	GTCCGGCGTTAAGTTATGGC	CGCCTGCGAGTTAACGATC
MLKL	GCTCAGGAAGAATGAATGC	GCCTTACTAGTCCAATGTCGC

### Statistical Analysis

Statistical analysis was conducted using unpaired Student’s *t* tests or by two-way ANOVA in GraphPad Prism 5 software.

## Results

### PRV Induces a Necrotic Form of Cell Death

**FIGURE 1:**
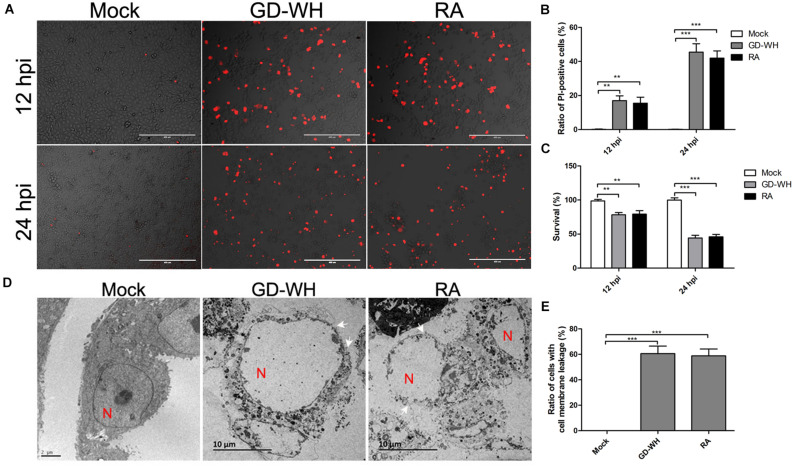
PRV induced cell death with necrotic characteristics. **(A)** PI-positive PK-15 cells were increased by PRV infection. PK-15 cells were mock-infected or infected with PRV GD-WH or RA strains (MOI = 10) for 12 and 24 h. After cells were stained with PI (red), live images were snapped. **(B)** Statistical analysis of the ratio of PI-positive cells in live images (mean ± SD; *n* = 3; **P < 0.01, ***P < 0.001). *P* values were calculated by using two-way ANOVA. **(C)** Cell viability was decreased by PRV infection. PK-15 cells were mock-infected or infected with PRV GD-WH or RA strains (MOI = 10) for 24 h. Cell viability was analyzed by using the CCK8 assay as described in the Materials and Methods section (mean ± SD; *n* = 3; **P < 0.01, ***P < 0.001). *P* values were calculated by using two-way ANOVA. **(D)** Disrupted membrane of PRV-infected cells was observed by using TEM. PK-15 cells in 10-cm dishes were mock-infected or PRV-infected at an MOI of 10 for 24 h. Then, cell monolayer was collected and analyzed by using TEM. In TEM images, N indicated the cell nucleus. Arrowheads point to cell plasma membrane leakage. **(E)** Quantification ratio of PK-15 with the disrupted cell plasma membrane (mean ± SD; *n* ≥ 30 cells; ***P < 0.001). *P* values were calculated by using an unpaired Student’s *t*-test.

PK-15 cells were susceptible to PRV infection *in vitro*. To analyze the cell death induced by PRV infection, PK-15 cells were stained with PI at 12 and 24 h post-infection (hpi). As shown in Figures 1A,B, PK-15 cells infected with PRV GD-WH or RA strains showed morphological changes, and the proportion of PI-positive live cells increased at 12 and 24 hpi, but the mock-infected cells were not affected. In addition, cell viability was sharply reduced by PRV GD-WH or RA infection at 12 and 24 hpi (Figure 1C). Considering that PI is a membrane-impermeable nuclear stain, this result showed that PRV infection caused the cell death by a membrane permeability change. To display cell membrane leakages of PK-15 cells infected by PRV, we observed the cell micromorphology by using transmission electron microscopy (TEM). Remarkably, disrupted cell membranes were observed in the PRV GD-WH or RA infection groups compared with normal cell morphology in the mock groups at 24 hpi, demonstrating the characteristics of necrotic cells. Meanwhile, swollen nuclei and dissolved chromatin were observed in PRV-infected cells (Figures 1D,E). These results indicated that both PRV GD-WH and RA strains induced necrotic cell death in PK-15 cells, independent of viral strain differences.

### Cell Death During PRV Infection Is Caspase Independent

**FIGURE 2:**
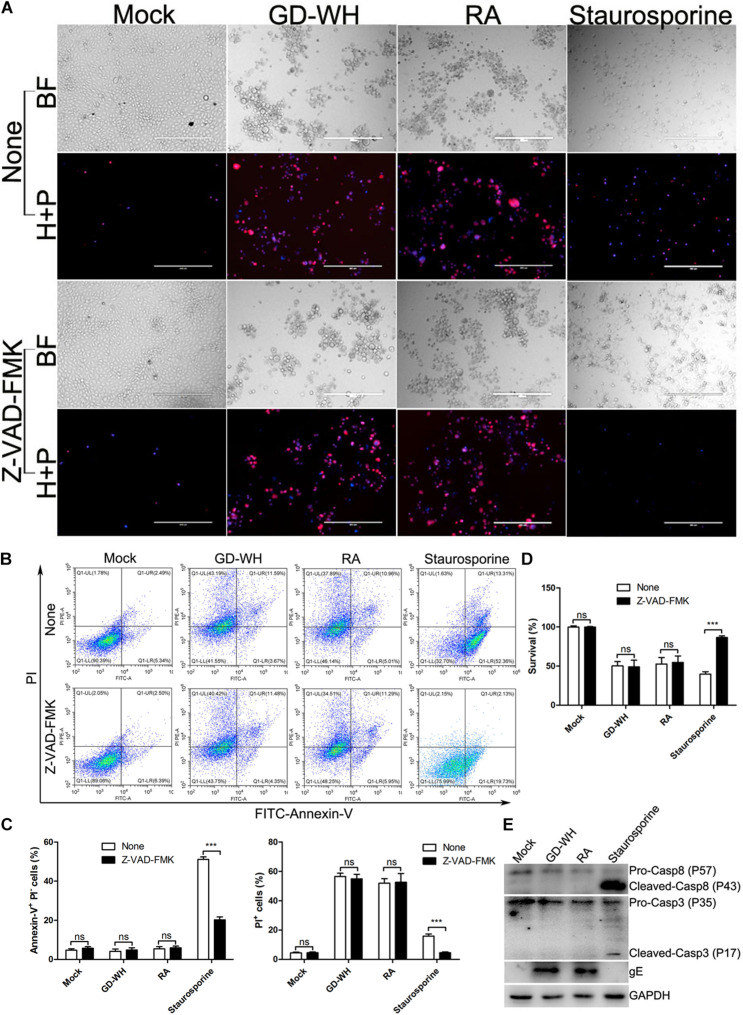
Cell death induced by PRV infection was caspase independent. **(A)** Z-VAD-FMK treatment has no inhibitory effect on increased PI-positive cells during PRV infection. PK-15 cells pretreated with 10 μM Z-VAD-FMK were mock-infected or infected with PRV GD-WH or RA strains (MOI = 10) for 24 h. After cells were stained with Hoechst-33342 (blue) and PI (red), live images were snapped. BF indicated the bright field images. H + P indicated the merged images of cells stained with Hoechst-33342 (blue) and PI (red). **(B)** Flow cytometry analysis of the effect of Z-VAD-FMK treatment on PRV-infected PK-15 cells. PK-15 cells pretreated with 10 μM Z-VAD-FMK were mock-infected or infected with PRV GD-WH or RA strains (MOI = 10) for 24 h. Rates of apoptotic cells were analyzed by using the Alexa Fluor^TM^ 488 Annexin V/Dead Cell Apoptosis Detection Kit as described in the Materials and Methods section (Q1-UL, Annexin V-negative and PI-positive; Q1-UR, Annexin V-positive and PI-positive; Q1-LL, Annexin V-negative and PI-negative; Q1-LR, Annexin-V-positive and PI-negative). **(C)** Statistical analysis of the ratio of apoptotic cells (Annexin-V-positive and PI-negative) or dead cells (PI-positive) in flow cytometry analysis data (mean ± SD; *n* = 3; *^*NS*^P* > 0.05, ***P < 0.001). *P* values were calculated by using two-way ANOVA. **(D)** The decline of cell viability induced by PRV infection cannot be recovered by Z-VAD-FMK treatment. PK-15 cells pretreated with 10 μM Z-VAD-FMK were mock-infected or infected with PRV GD-WH or RA strains (MOI = 10) for 24 h. Cell viability was analyzed by using the CCK8 assay as described in the Materials and Methods section (mean ± SD; *n* = 3; *^*NS*^P* > 0.05, ***P < 0.001). *P* values were calculated by using an unpaired Student’s *t*-test. **(E)** Western blot analysis showed that caspase proteins were not activated during PRV infection. PK-15 cells were mock-infected, infected with PRV GD-WH or RA strains (MOI = 10), or 50 nM staurosporine treated for 24 h. Caspase-3 and caspase-8 in whole cell lysates were analyzed by using Western blot as described in the Materials and Methods section.

To exclude the possibility that necrotic cells in the PRV-infected groups were linked to caspase-dependent cell death, the pan-caspase inhibitor Z-VAD-FMK was utilized to globally inhibit all known caspases before viral infection. Live images showed that staurosporine, an activator of apoptosis, increased the number of apoptotic cells stained with Hoechst-33342, a membrane-permeable nuclear stain. Although Z-VAD-FMK treatment obviously reduced the effect of staurosporine stimulation, it had no effect on PRV GD-WH or RA strains inducing necrotic cells stained with both Hoechst-33342 and PI (Figure 2A). Meanwhile, flow cytometry analysis also indicated that increased ratios of necrotic cells (PI-positive) in the PRV GD-WH or RA infection groups were not affected by Z-VAD-FMK treatment, but Z-VAD-FMK had obvious inhibitory effect on ratios of apoptotic cells (Annexin V-positive and PI-negative) stimulated by staurosporine (Figures 2B,C). Further experiments showed that the decline of cell viability induced by staurosporine stimulation could be reversed by Z-VAD-FMK treatment, but not by PRV GD-WH or RA infection (Figure 2D). In addition, Western blot analysis indicated that cleaved caspase-3 and caspase-8 were not detected in the PRV-infected groups at 24 hpi (Figure 2E). These results offered evidence that caspase activities were not involved in PRV GD-WH or RA strain induction of necrotic cell death.

### Classical RIPK1-Dependent Necroptosis Is Not Related to PRV Infection

**FIGURE 3:**
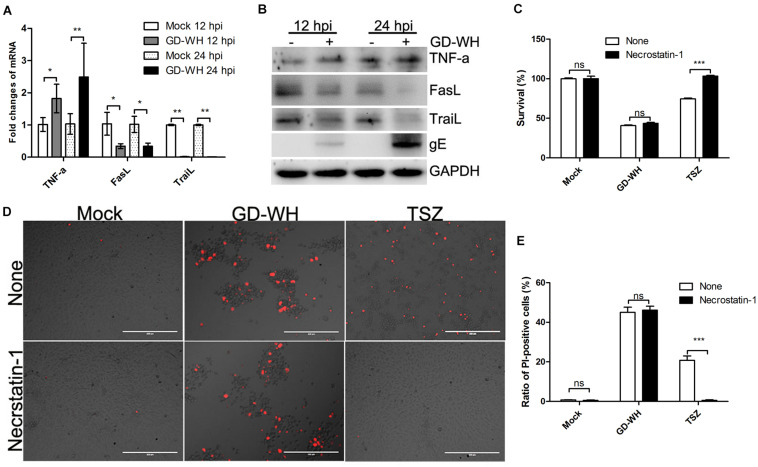
Necrotic cell death during PRV infection was not related to RIPK1. **(A)** PRV infection raised the TNF-α transcription. PK-15 cells were mock-infected or infected with PRV GD-WH strain (MOI = 10) for 24 h. mRNA levels of TNF-α, FasL, and TraiL were analyzed by RT-qPCR as described in the Materials and Methods section (mean ± SD; *n* = 3; *P < 0.05, **P < 0.01). *P* values were calculated by using two-way ANOVA. **(B)** Western blot analysis protein expressions of TNF-α, FasL, and TraiL. PK-15 cells were mock-infected or infected with PRV GD-WH strain (MOI = 10) for 24 h. Protein expressions of TNF-α, FasL, and TraiL in whole cell lysates were analyzed by using Western blot as described in the Materials and Methods section. **(C)** Necrostatin-1 could not prevent the decline of cell viability during PRV infection. PK-15 cells pretreated with 100 μM necrostatin-1 were mock-infected or infected with PRV GD-WH strain (MOI = 10) for 24 h. Cell viability was analyzed by using the CCK8 assay as described in the Materials and Methods section (mean ± SD; *n* = 3; *^*NS*^P* > 0.05, ***P < 0.001). *P* values were calculated by using an unpaired Student’s *t*-test. **(D)** Necrostatin-1 had no effect on necrotic cells with PI staining induced by PRV infection. PK-15 cells pretreated with 100 μM necrostatin-1 were mock-infected or infected with PRV GD-WH strain (MOI = 10) for 24 h. PK-15 cells treated with TSZ (10 ng/ml TNF-α combined with 2 μM SM-164 and 10 μM Z-VAD-FMK) were used as a positive control of classical necroptosis. After cells were stained with PI (red), live images were snapped. **(E)** Statistical analysis of the ratio of PI-positive cells in live images (mean ± SD; *n* = 3; *^*NS*^P* > 0.05, ***P < 0.001). *P* values were calculated by using an unpaired Student’s *t*-test.

Based on the fact that both PRV GD-WH and RA strains induced caspase-independent necrotic cell death in PK-15 cells, we further explored how PRV induced necrotic cell death by using the GD-WH strain. Classical necroptosis is activated by the interaction of TNF-α with death receptors and RIPK1 (Holler et al., 2000). To explore the role of classical necroptosis in PRV infection, the transcription of TNF-α was analyzed by RT-qPCR. Meanwhile, the transcription levels of FasL and TraiL were measured in parallel, as these are ligands corresponding to death receptors of the extrinsic apoptosis pathway. As shown in Figure 3A, PRV GD-WH infection raised the TNF-α transcription level in a time-dependent manner but decreased the transcription of FasL and TraiL. Further analysis showed that the protein expression of TNF-α, FasL, and TraiL was consistent with transcriptional changes in PK-15 cells infected by PRV GD-WH (Figure 3B). These results suggested that RIPK1 might be activated by TNF-α during PRV infection. Then, we asked whether the specific RIPK1 inhibitor necrostatin-1 could prevent the cell death induced by PRV GD-WH infection. Surprisingly, necrostatin-1 failed to reverse the cell viability decline in the PRV GD-WH infection group, but successfully restored the cell viability in the group treated with TNF-α/Smac mimetic/Z-VAD-FMK, a classical necroptosis activator (Figure 3C). The live images showed that necrostatin-1 could reduce the ratio of PI-positive cells induced by TNF-α/Smac mimetic/Z-VAD-FMK treatment while having no effect on necrotic cells induced by PRV GD-WH infection (Figures 3D,E). Taken together, these results suggested that TNF-α- and RIPK1-dependent necroptosis was not involved in PRV infection.

### PRV Infection Upregulates the Phosphorylation of RIPK3 and MLKL

**FIGURE 4:**
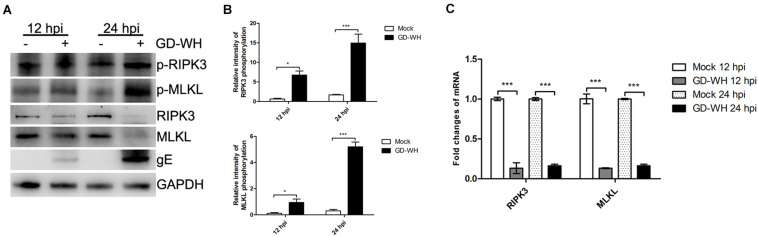
PRV enhanced the phosphorylation of RIPK3 and MLKL. **(A)** Western blot analysis showed that the phosphorylation of RIPK3 and MLKL was increased by PRV infection. PK-15 cells were mock-infected or infected with PRV GD-WH strain (MOI = 10) for 24 h. The phosphorylation or protein expression of RIPK3 and MLKL in whole cell lysates was analyzed by using Western blot as described in the Materials and Methods section. **(B)** Statistical analysis of the intensity of phosphorylation bands. Relative intensity of RIPKK3 or MLKL phosphorylation was obtained by comparing the densitometry of phosphorylation bands to its protein expression bands. GAPDH was used as an internal loading control (mean ± SD; *n* = 3; *P < 0.05, ***P < 0.001). *P* values were calculated by using two-way ANOVA. **(C)** PRV inhibited the transcription of RIPK3 and MLKL. PK-15 cells were mock-infected or infected with PRV GD-WH strain (MOI = 10) for 12 or 24 h. mRNA levels of RIPK3 and MLKL were analyzed by RT-qPCR as described in the Materials and Methods section (mean ± SD; *n* = 3; ***P < 0.001). *P* values were calculated by using two-way ANOVA.

In addition to RIPK1, it has been reported that the necroptosis pathway can be activated by the direct reaction of some PRRs with RIPK3 (Kaiser et al., 2013). Although RIPK1-dependent necroptosis was not related to PRV infection, we asked whether RIPK3 and downstream MLKL were activated in a RIPK1-independent manner. Western blot analysis showed that the phosphorylation of RIPK3 and MLKL was increased by PRV GD-WH infection in PK-15 cells at 12 and 24 hpi. Meanwhile, the protein expression levels of RIPK3 and MLKL were downregulated by GD-WH at 12 and 24 hpi (Figures 4A,B). In addition, the transcription of RIPK3 and MLKL was analyzed. As shown in Figure 4C, RT-qPCR analysis indicated that the transcription levels of RIPK3 and MLKL were sharply downregulated in PK-15 cells infected by PRV GD-WH, consistent with changes of the respective protein expression levels. Above all, these results demonstrated that RIPK3- and MLKL-dependent necroptosis was activated in PRV-infected PK-15 cells.

### Knockdown of RIPK3 and MLKL Reduces Necroptosis but Enhances Viral Replication in PRV-Infected PK-15 Cells

**FIGURE 5:**
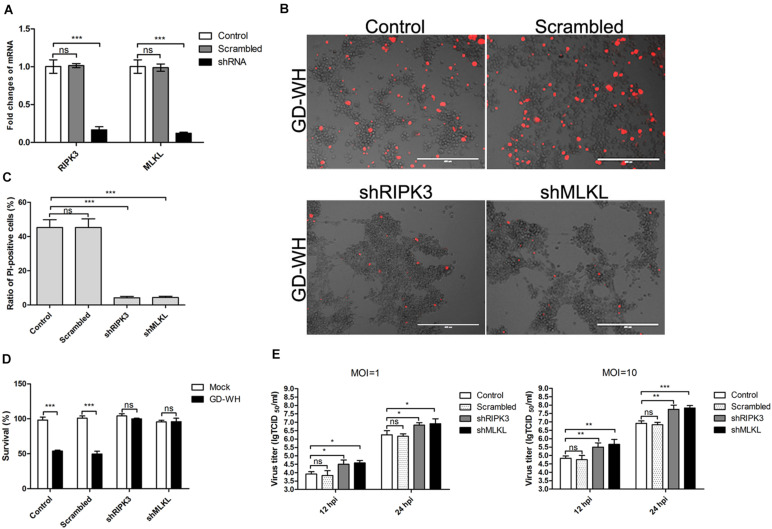
Knockdown of RIPK3 and MLKL dampened PRV-induced necroptosis but increased viral titers. **(A)** qPCR analysis indicated that RIPK3 or MLKL transcription was down-regulated in PK-15 cells stably expressing shRNA. mRNA levels of RIPK3 and MLKL were analyzed by RT-qPCR as described in the Materials and Methods section (mean ± SD; *n* = 3; *^*NS*^P* > 0.05, ***P < 0.001). *P* values were calculated by using two-way ANOVA. **(D)** Knockdown of RIPK3 or MLKL prevented the reduction of cell viability during PRV infection. PK-15 cells stably expressing shRNA were mock-infected or infected with PRV GD-WH strain (MOI = 10) for 24 h. Cell viability was analyzed by using the CCK8 assay as described in the Materials and Methods section (mean ± SD; *n* = 3; *^*NS*^P* > 0.05, ***P < 0.001). *P* values were calculated by using an unpaired Student’s *t*-test. **(B)** RIPK3 or MLKL knockdown decreased PI-positive cells during PRV infection. PK-15 cells stably expressing shRNA were mock-infected or infected with PRV GD-WH strain (MOI = 10) for 24 h. After cells were stained with PI (red), live images were snapped. **(C)** Statistical analysis of the ratio of PI-positive cells in live images (mean ± SD; *n* = 3; *^*NS*^P* > 0.05, ***P < 0.001). *P* values were calculated by using an unpaired Student’s *t*-test. **(E)** Virus titers were increased in RIPK3 or MLKL knockdown PK-15 cells. PK-15 cells stably expressing shRNA were mock-infected or infected with PRV GD-WH strain (MOI = 1 or 10) for 12 or 24 h. Virus titers were analyzed as described in the Materials and Methods section (mean ± SD; *n* = 3; *^*NS*^P* > 0.05, *P < 0.05, **P < 0.01, ***P < 0.001). *P* values were calculated by using two-way ANOVA.

An RNA interference experiment was performed to verify the functions of RIPK3 and MLKL in PRV-induced necroptosis. The RT-qPCR analysis indicated that PK-15 cells stably expressing shRNA targeted to RIPK3 or MLKL displayed an obvious decline of their transcription products (Figure 5A). After RIPK3 or MLKL knockdown, PK-15 cells were infected by PRV GD-WH at 24 hpi, only a few PI-positive cells were observed compared with the mock and scrambled groups (Figures 5B,C). In addition, our experiments showed that knockdown of RIPK3 or MLKL could reverse the reduction of cell viability in the PRV-infected groups (Figure 5D). Considering this, the viral replication in RIPK3 or MLKL knockdown PK-15 cells was further investigated. Interestingly, we found that viral titers at 12 and 24 hpi were both increased in PK-15 cells with RIPK3 or MLKL gene knockdown, independent of the multiplicity of infection (MOI) of PRV infection (Figure 5E). These results indicated that RIPK3 or MLKL was essential for PRV-induced necroptosis and was a limiting factor for viral replication in host cells.

## Discussion

Pseudorabies virus is an alpha-herpesvirus with the ability to cause necrosis in the central nervous system, lung, kidney, and other organs (Sehl and Teifke, 2020). During its invasion metastasis *in vivo*, virus replication and lytic infection in epithelial cells are crucial events (Nauwynck et al., 2007). In this study, we provided the first evidence that necroptosis was involved in PRV-induced cell death in PK-15 cells, a porcine kidney epithelial cell line. Necroptosis has been reported to severely impair tissue necrosis, cause inflammation, and limit *Vaccinia* virus replication (Cho et al., 2009). Necroptosis is related to monocyte cell death during influenza A virus infection, subsequently initiating adaptive immunity response and inflammation (Lee et al., 2019). Herein, we speculated that PRV-induced necroptosis might be a key link between virus lytic infection in epithelial cells and necrotic inflammatory response *in vivo*. Conversely, the RHIM-containing protein of HSV-1, ICP6, reduced necroptosis in human cells (Guo et al., 2015b). Although PRV is closely related to HSV-1, the role of necroptosis in viral pathology might be different in its natural host.

Unlike apoptosis, necroptosis will lead to cell membrane rapture and cellular content leakage (Wang H et al., 2014). The leakage of dying cells is the initiator of adaptive immunity response and inflammation (Nailwal and Chan, 2019). Indeed, cellular content leakage in PK-15 cells infected by PRV was observed in the TEM analysis. Our results also showed that inhibition of necroptosis could prevent cell death and enhance PRV replication in PK-15 cells. Therefore, we suggest that initiation of necroptosis in PRV-infected cells is possibly a protective mechanism against viral replication in host cells. However, the leakage of virus-infected cells causes inflammation at the viral invasion site, a factor that is related to the necrotic pathology in multiple organs in PRV-infected pigs. Remarkably, apoptosis is a programmed cell death without cellular content leakage and inflammation *in vivo* (Yatim and Albert, 2011). It has been demonstrated that herpes virus employs specific strategies to escape clearance by apoptosis of infected cells (Guo et al., 2015a). Additionally, we showed that the pan-caspase inhibitor had no effect on the cell death caused by PRV infection. Caspase proteins were not activated in PRV-infected PK-15 cells. This excluded the role of caspase-dependent cell death pathways in PRV infection. As for PRV, it has been shown that the US3 protein mainly exerts the anti-apoptotic function (Geenen et al., 2005; Chang et al., 2013).

Concerning mechanism, we found that the transcription of TNF-α was increased in PRV-infected PK-15 cells, whereas classical RIPK1 was not related to necroptosis during viral infection. However, the phosphorylation of RIPK3 and MLKL offers direct evidence that necroptosis was occurring in the infectious process. Furthermore, the effect of RIPK3 and MLKL knockdown on cell death demonstrated their essential roles in virus-induced necroptosis, although the transcription levels of RIPK3 and MLKL were downregulated by virus infection. As a defensive mechanism limiting viral replication in host cells, necroptosis is well known to be activated by RIPK1 or ZBP1 (Wu et al., 2014; Thapa et al., 2016). *Vaccinia* virus-infected cells were sensitive to TNF-α- and RIPK1-mediated necroptosis but not responsive to ZBP1 (Li and Beg, 2000; Koehler et al., 2017). In contrast, influenza virus activates necroptosis by ZBP1 sensing viral RNA (Thapa et al., 2016). Remarkably, MCMV or HSV-1 can inhibit necroptosis in natural host cells *via* encoding viral proteins with an inhibitory RHIM targeted to RIPK1 and ZBP1 (Upton et al., 2012; Guo et al., 2015b). As an animal herpes virus linked to HSV-1, PRV showed a special necroptosis process different from HSV-1 in its natural host cells. Notably, it has been reported that HSV-1 can directly initiate necroptosis by ICP6 binding to RIPK3 in mouse cells (Wang X et al., 2014). Deletion of the RHIM of ICP6 will result in necroptosis of human cells infected by HSV-1 (Guo et al., 2018). We speculated that ICP6 might be responsible for the different effects on necroptosis between HSV-1 and PRV infection in their natural host cells. However, this needs to be further explored in future studies.

In conclusion, our data are the first demonstration of RIPK3-dependent necroptosis in PRV-infected porcine cells. Meanwhile, we found that necroptosis of host cells played a limiting role in PRV infection. Considering that necroptosis is a type of programmed cell death related to cellular leakage and inflammation, we believe that our data will be beneficial for understanding the necrotic pathology during PRV infection *in vivo*. Further study focused on verifying the role of necroptosis *in vivo* by using RIPK3-knockout mice will be necessary. Inhibitors targeting RIPK3 and MLKL in the necroptosis pathway may someday be helpful for controlling PRV infection and exerting a curative effect.

## Data Availability Statement

The original contributions presented in the study are included in the article/supplementary material, further inquiries can be directed to the corresponding author/s.

## Author Contributions

HG carried out the data analysis and drafted the manuscript. ZB, PC, and SS participated in the experiments. ZJ, YL, and KZ participated in the data analysis. RC and CL conceived the study. DY prepared the materials for the experiments. All the authors read and approved the final manuscript.

## Conflict of Interest

The authors declare that the research was conducted in the absence of any commercial or financial relationships that could be construed as a potential conflict of interest.
